# Evaluation of factors influencing xanthelasma palpebrarum in Chinese patients: A case-control study

**DOI:** 10.1097/MD.0000000000043325

**Published:** 2025-07-18

**Authors:** Zhen-Xing Liu, Ting-Ting Zhu, Dan Huang, Ting Xi

**Affiliations:** a Department of Ophthalmology, Suzhou Hospital Affiliated Nanjing Medical University, Suzhou, Jiangsu, China.

**Keywords:** blood glucose, blood type, body mass index, dyslipidemia, xanthelasma palpebrarum

## Abstract

Xanthelasma palpebrarum (XP) is a yellow plaque lesion near the eyelid, which was previously found to be associated with dyslipidemia, metabolic syndrome, and cardiovascular disease. This study aimed to explore the clinical characteristics and potential mechanisms underlying its development by analyzing body mass index (BMI), lipid levels, blood glucose, and blood type in affected individuals. A case-control study was conducted involving 44 patients with XP between 2019 and 2023 and 44 age- and sex-matched healthy controls. Data collection included medical history, BMI, total cholesterol (TC), triglycerides (TG), blood glucose, and blood type. Patients were predominantly female (68.2%) and aged 45–50 years. Compared with healthy controls, patients exhibited significantly higher mean levels of TC and TG (*P* = .01 and *P* = .04, respectively). Elevated TC and TG levels were more common in the XP group (TC: 50.0% vs 15.91%; TG: 25.0% vs 6.82%). Hyperglycemia was also more frequent (15.91% vs 0%, *P* < .05). However, there were no significant differences in mean BMI, blood glucose levels, or overweight prevalence. Blood type distributions were similar between the 2 groups, and no statistically significant differences were found. Multivariate logistic regression analysis revealed that TC, TG, BMI, and blood glucose were not found as independent risk factors for XP. The findings suggest that while dyslipidemia is associated with XP, its development is likely due to the combined influence of lipid abnormalities and genetic predisposition rather than any single clinical factor.

## 1. Introduction

Xanthelasma palpebrarum (XP) is a common skin lesion characterized by yellowish or orange flat plaques, typically located on the medial aspect of the upper eyelid. Although painless, XP mainly influences facial aesthetics, prompting patients to seek treatment.^[1]^ Traditionally known as a localized skin condition, emerging evidence suggests a strong association between XP and systemic metabolic disorders, particularly dyslipidemia. Studies have demonstrated that XP patients frequently exhibit lipid abnormalities, such as hypercholesterolemia and hypertriglyceridemia, which are core risk factors for atherosclerosis and cardiovascular disease.^[2,3]^ Notably, dyslipidemia not only promotes vascular endothelial injury and lipid deposition, but may also mediate XP formation through local inflammatory responses. In addition, body mass index (BMI), as a key indicator for obesity assessment, has been reported to be significantly correlated with insulin resistance, hypertension, and impaired glucose and lipid metabolism, further suggesting that metabolic syndrome may serve as a common pathological basis for XP and cardiovascular disease.^[4]^ Hyperglycemia exacerbates vascular injury by inducing oxidative stress and endothelial dysfunction, while elevated levels of triglycerides (TG) promote atherogenic lipoprotein accumulation.^[5]^ Abnormalities in these metabolic parameters not only reflect the systemic imbalance, but also provide valuable indicators for assessing cardiovascular risk in XP patients. However, the majority of XP patients concentrate solely on cosmetic concerns, mainly overlooking underlying systemic risks, leading to missed opportunities for early intervention. Although international studies have preliminarily investigated the association between XP and metabolic disorders, evidence for the Chinese population remains scarce. In addition, previous studies have mostly examined isolated metabolic parameters without adequately adjusting for confounding variables, such as age and sex, thereby reducing the strength of their conclusions. A comprehensive analysis of the association of XP with BMI, total cholesterol (TC), TG, and blood glucose is thus essential to clarify its pathogenesis and promote more effective clinical management.

## 2. Methods

### 2.1. Study population

This retrospective case-control study included 44 patients with XP who were diagnosed in the Department of Ophthalmology of Suzhou Hospital Affiliated Nanjing Medical University between January 2019 and December 2023 as the study group. To control for potential confounding factors, 44 age- and sex-matched healthy subjects without XP were randomly selected from participants attending during the same period to form the control group. All participants signed informed consent forms, and the study protocol was reviewed and approved by the Ethics Committee of our hospital. Exclusion criteria were applied to minimize interference from metabolic and systemic diseases, including abnormal liver or kidney function (alanine aminotransferase/aspartate aminotransferase >2 × the upper limit; estimated glomerular filtration rate <60 mL/min/1.73 m^2^), thyroid dysfunction (thyroid stimulating hormone outside the 0.4–4.0 mIU/L range), inflammatory skin diseases (e.g., psoriasis, lupus erythematosus), and recent use (within 6 months) of lipid-lowering agents or hormonal therapies. Demographic characteristics (age and sex) and metabolic parameters, including BMI, blood glucose, TC, and TG, were standardized and collected by an electronic medical record system. All laboratory tests were conducted in the hospital’s clinical laboratory following standardized procedures.

### 2.2. Study methods

Standardized physical examinations and biochemical tests were performed by professional nurses in this study. Anthropometric measurements included height, recorded to the nearest 0.1 cm using a calibrated stadiometer (with participants standing upright, shoes and headwear removed), and weight, measured to the nearest 0.1 kg using a uniformly calibrated electronic scale (with participants wearing light clothing and no shoes or headwear). BMI was calculated as weight (kg) divided by height squared (m^2^). According to the criteria established by the Chinese Obesity Task Force,^[4]^ BMI was classified into 4 categories: underweight (<18.5×kg/m^2^), normal (18.5–23.9 kg/m^2^), overweight (24.0–27.9 kg/m^2^), and obese (≥28.0 kg/m^2^). For biochemical testing, fasting venous blood samples were collected in the morning to measure blood glucose, TC, and TG using certified equipment approved by the metrology department. Diagnostic thresholds were based on international consensus guidelines^[5,6]^: hypercholesterolemia was defined as TC ≥ 5.2 mmol/L, hypertriglyceridemia as TG ≥ 1.69 mmol/L, and hyperglycemia as fasting blood glucose ≥ 6.1 mmol/L.

### 2.3. Statistical analysis

SPSS 24.0 software (IBM, Armonk, NY) was used for statistical analysis. Continuous variables were described as mean ± standard deviation, and paired sample *t* test was employed for making comparisons between groups according to age/sex matching characteristics. Categorical variables were expressed as frequency (percentage), and differences between groups were assessed by *χ*^2^ test or the Fisher exact test. Independent risk factors for XP were identified using multivariate logistic regression analysis, including BMI, TC, TG, and blood glucose as variables. The strength of association was expressed as odds ratio with 95% confidence interval. Statistical analysis was performed using 2-sided tests, and statistical significance was set at *P* < .05.

## 3. Results

### 3.1. Demographic characteristics and baseline data

**Figure 1.**
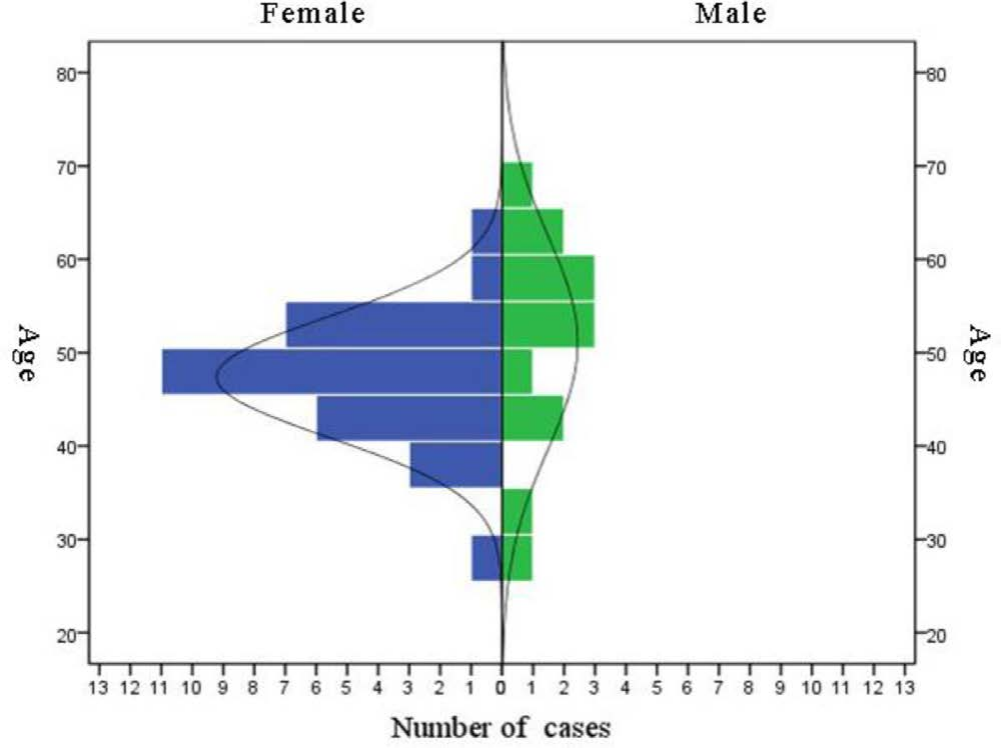
Population structure diagram in the 2 groups.

**Figure 2.**
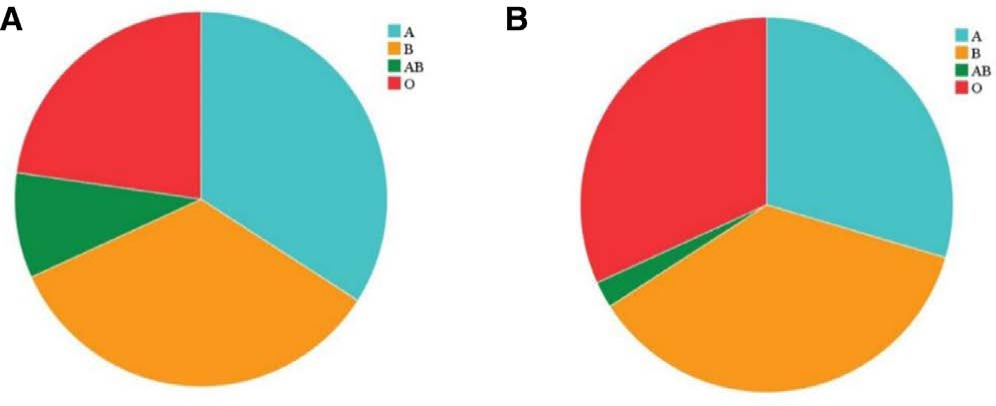
Distribution of blood types in the 2 groups. A represents the study group, and B indicates the control group.

The study included 44 patients with XP and 44 age- and sex-matched healthy controls. Gender analysis revealed 14 (31.82%) men and 30 (68.18%) women in the study group, indicating a significantly higher proportion of women (*χ*^2^ = 5.82, *P* = .016). Patients’ age in the study group ranged from 28 to 67 years, with a mean age of 48.57 ± 8.43 years. The 45–50 age group had the highest representation (13 cases, 29.55%) (Fig. 1). The proportions of blood types A, B, AB, and O in the study group were 34.09%, 34.09%, 9.09%, and 22.73%, respectively, while in the control group, the corresponding proportions were 29.55%, 36.36%, 2.27%, and 31.82% (Fig. 2). No statistically significant difference was found in blood type distribution between the 2 groups (*χ*^2^ = 2.642, *P* = .45).

### 3.2. Comparison of BMI between the 2 groups

Comparison of BMI distribution between the 2 groups indicated that the overweight rate (BMI ≥ 24 kg/m^2^) in the study group was 47.7% (21/44), which was 15.9% higher than that in the control group (31.8%, 14/44). Although there was a trend of clinical difference, Pearson *χ*^2^ test indicated that there was no statistically significant difference between the 2 groups (*χ*^2^ = 2.325, *P* = .127) (Table 1).

**Table 1:** Comparison of BMI between the 2 groups.

Group	BMI ≥ 24 kg/m^2^	BMI < 24 kg/m^2^	Total
Study group	21 (47.7%)	23 (52.3%)	44
Control group	14 (31.8%)	30 (68.2%)	44

BMI = body mass index.

### 3.3. Comparison of TC, TG, blood glucose, and BMI between the 2 groups

**Figure 3.**
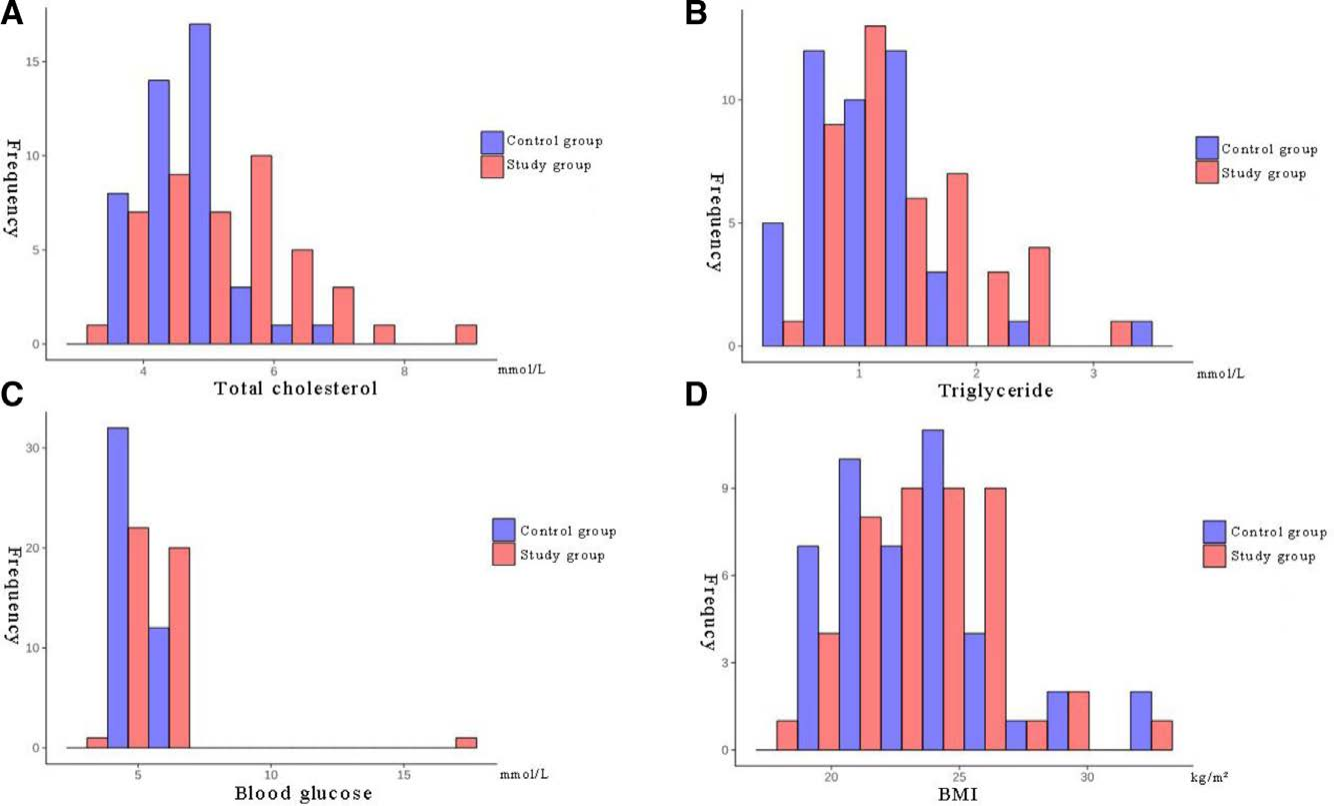
Histograms illustrating the distributions of metabolic parameters in the 2 groups. (A) Total cholesterol; (B) triglycerides; (C) blood glucose; (D) BMI. BMI = body mass index.

Comparison of the abnormal rates of metabolic parameters revealed that the proportion of elevated TC level (≥5.2 mmol/L) was significantly higher in the study group than that in the control group (50.00% [22/44] vs 15.91% [7/44]; *χ*^2^ = 11.47, *P* < .001). Similar trends were noted in the abnormal levels of TG (≥1.69 mmol/L) (25.00% [11/44] vs 6.82% [3/44]; *χ*^2^ = 5.94, *P* = .015, odds ratio = 4.56, 95% confidence interval: 1.21–17.21), while the rate of abnormal blood glucose (blood glucose ≥ 6.1 mmol/L) was 15.91% [7/44] in the study group and no abnormalities were detected in the control group (Fisher exact test, *P* = .006) (Fig. 3). Comparison of mean continuous variables revealed that the levels of TC and TG were significantly higher in the study group than those in the control group, while blood glucose and BMI exhibited an increasing trend, however, the difference did not reach statistical significance (Table 2).

**Table 2:** Comparison of total cholesterol, triglyceride, blood glucose, and BMI between the 2 groups.

Group	Total cholesterol (mmol/L)	Triglycerides (mmol/L)	Blood glucose (mmol/L)	BMI (kg/m^2^)
Study group	5.20 ± 1.19	1.36 ± 0.62	5.65 ± 1.97	23.76 ± 2.86
Control group	4.68 ± 0.62	1.10 ± 0.56	5.10 ± 0.41	23.29 ± 3.31
*t*	2.56	2.06	1.80	0.71
*P* value	.01*	.04*	.08	.48

BMI = body mass index.

* *P* value < .05.

### 3.4. Analysis of independent risk factors for XP

**Figure 4.**
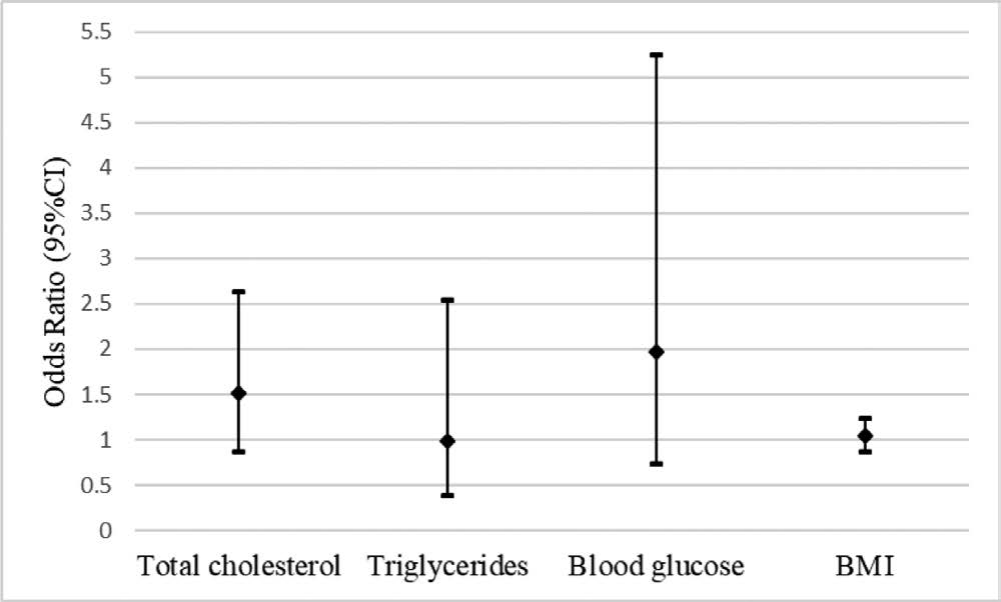
The association between XP and metabolic parameters. CI indicates confidence interval, and all CIs spanned the null value (1.0). BMI = body mass index, XP = xanthelasma palpebrarum.

Multivariate logistic regression analysis revealed that after adjusting for age, gender, and other metabolic parameters, the regression coefficients of BMI (β = 0.039/kg/m^2^, Wald *χ*^2^ = 0.19, *P* = .666), TC (β = 0.411/mmol/L, *P* = .148), TG (β = ‐0.012/mmol/L, *P* = .980), and fasting blood glucose (β = 0.680/mmol/L, *P* = .172) did not reach the threshold of statistical significance (Fig. 4). The combined model results indicated that BMI, TC, TG, and blood glucose were not identified as independent risk factors for the development of XP in a strictly age- and sex-matched case-control design (all *P* > .05).

## 4. Discussion

XP is commonly presented in clinical settings due to cosmetic concerns. However, patients mainly overlook the potential underlying metabolic abnormalities associated with the condition. In this study, the majority of XP patients were characterized by sporadic dyslipidemia, specifically elevated TC and TG levels. A significant proportion of affected cases were women who aged 45–50 years, which may reflect a gender-based tendency to seek medical attention for aesthetic concerns.^[7,8]^ Importantly, TC, TG, blood glucose, and BMI did not appear as independent risk factors for XP. These findings align with those of Yael Lustig et al,^[9]^ whose large population-based study similarly found no significant association between XP, dyslipidemia, or cardiovascular disease, thereby challenging the function of XP as a reliable marker for systemic risk.

Although studies have suggested an association between dyslipidemia and XP,^[10–12]^ 50% of patients in this study had normal lipid levels, demonstrating that the relationship between them remains elusive. Ma Teng et al found that abnormal metabolism of lipoprotein(a) was common in patients with XP, suggesting that it may account for the occurrence of normolipidemia in some cases.^[13,14]^ However, lipoprotein(a) levels are largely genetically determined, minimally influenced by environmental or lifestyle factors, and tend to remain stable throughout adulthood.^[15]^ Therefore, lipoprotein(a) alone cannot fully explain the dyslipidemia found in XP patients. Genetic studies provide further insights into this discrepancy. Wang Zhi-min et al excluded a link between XP and exon mutations of the *LDLR* gene or familial hypercholesterolemia and demonstrated that high-density lipoprotein level might be implicated in XP pathogenesis.^[16]^ Similarly, Li Yong-rong et al found no significant differences in single nucleotide polymorphisms of macrophage scavenger receptor 1 (*MSR1*), a gene related to atherosclerosis, between XP patients and healthy controls. However, homozygous mutations at specific MSR1 exon loci were identified in patients with both XP and coexisting carotid atherosclerosis and hyperlipidemia.^[17]^ A recent Mendelian randomization study further highlighted genetic contributors to XP, revealing significant associations with TC, low-density lipoprotein cholesterol, and 3 inflammation-related proteins, emphasizing the dual role of lipid metabolism and inflammatory responses in XP development.^[18]^ Taken together with existing literature,^[16–19]^ a hierarchical regulatory hypothesis was proposed in this study: an individual’s genetic threshold for lipid tolerance may determine susceptibility to XP. The lesion may develop when metabolic imbalance exceeds the compensatory capacity of genetic regulatory mechanisms. This hypothesis warrants further validation through genomic approaches, such as genome-wide association studies, to identify regulatory genes involved in lipid metabolism.

In addition to genetic and metabolic factors, age and gender have been identified as independent factors, and differences in incidence in different populations may be associated with hormones’ levels and skin aging.^[20,21]^ Previous studies have demonstrated that metabolic syndrome-related factors, such as lipid levels (e.g., TC, low-density lipoprotein, high-density lipoprotein, and TG), diabetes, and hypertension may all be associated with the development of XP. The lifestyle factors, such as smoking and alcohol consumption, may also increase the risk of XP by affecting lipid metabolism in vivo.^[3,22–25]^ In this study, a retrospective case-control design was employed to comprehensively analyze the differences in BMI, TC, TG, blood glucose, and blood type between XP patients and healthy controls on the basis of controlling for confounding factors, such as age and gender. The results not only provide evidence for the metabolic etiology theory of XP in the Chinese population, but also guide metabolic syndrome screening and individualized intervention for XP patients in clinical practice. This exploration may fill in the research gaps in relevant fields in China and improve patients’ and doctors’ perception of the systemic risks of XP.

## 5. Study limitation

This study has several limitations. Firstly, as an observational study, although most metabolic-related conditions were controlled, the influence of unmeasured confounding factors, such as dietary habits, ethnicity, and other lifestyle variables, could not be entirely ruled out and might partly account for some of the findings. Additionally, the study was conducted at a single center, which might introduce selection bias in the ophthalmology patient population. Some borderline results lacking statistical significance might also reflect limitations in sample size. Future research should consider long-term, prospective cohort designs with larger and more diverse populations to further elucidate the biological mechanisms underlying the association between XP and metabolic parameters.

## 6. Conclusion

Our study systematically identified several clinical factors associated with XP in Chinese patients, including age, sex, TC, and TG. However, BMI, blood glucose, and blood type demonstrated nonsignificant differences between groups. Multivariate analysis revealed that none of these parameters emerged as independent risk factors for XP. These findings support the multifactorial pathogenesis of XP, involving complex interactions between modifiable metabolic factors and inherent susceptibility. Notably, our results underscore the importance of recognizing XP lesions in specific demographic subgroups (particularly middle-aged females) as cutaneous markers of metabolic dysregulation, warranting proactive clinical monitoring of lipid profiles and systemic health.

## Author contributions

**Conceptualization:** Zhen-Xing Liu, Ting-Ting Zhu, Ting Xi.

**Data curation:** Zhen-Xing Liu.

**Investigation:** Zhen-Xing Liu, Ting-Ting Zhu.

**Methodology:** Zhen-Xing Liu, Dan Huang.

**Project administration:** Ting-Ting Zhu, Ting Xi.

**Resources:** Zhen-Xing Liu.

**Validation:** Zhen-Xing Liu, Ting-Ting Zhu, Ting Xi.

**Visualization:** Zhen-Xing Liu, Dan Huang.

**Writing – original draft:** Zhen-Xing Liu.

**Writing – review & editing:** Ting-Ting Zhu, Ting Xi.

## References

[1] Wang KY Hsu KC Liu WC Yang KC Chen LW. Relationship between xanthelasma palpebrarum and hyperlipidemia. Ann Plast Surg. 2018;80(2S Suppl 1):S84–6. 29424765 10.1097/SAP.0000000000001310

[2] Zhang RF Yang YW Gu JY Qi FZ. Xanthelasma palpebrarum and systemic diseases. Chin J Med Aesthet Cosmet. 2021;27:282–4.

[3] Jia H Qu XY Liu YP Lu Q Jiang X. Research progress on the relationship between xanthelasma palpebrarum and atherosclerotic cardiovascular disease. Chin J Evid Based Cardiovasc Med. 2018;10:252–3.

[4] China Obesity Working Group. Guidelines for prevention and control of overweight and obesity in Chinese adults (excerpt). Acta Nutr Sin. 2004;26:1–4.

[5] Chinese Diabetes Society. Guideline for the prevention and treatment of type 2 diabetes mellitus in China (2020 edition) (part 1). Chin J Pract Intern Med. 2021;41:668–95.

[6] Li JJ Zhao SP Gao RL. Chinese guidelines for lipid management (2023). Chin Circ J. 2023;38:237–71.

[7] Chang HC Sung CW Lin MH. Serum lipids and risk of atherosclerosis in xanthelasma palpebrarum: a systematic review and meta-analysis. J Am Acad Dermatol. 2020;82:596–605. 31499151 10.1016/j.jaad.2019.08.082

[8] Lousa R Alves M Mota C. The association of xanthelasma palpebrum with cardiovascular events: systematic review with meta-analysis. Eur J Prev Cardiol. 2022;29:1740–3. 35554519 10.1093/eurjpc/zwac070

[9] Lustig-Barzelay Y Kapelushnik N Goldshtein I. Association between xanthelasma palpebrarum with cardiovascular risk and dyslipidemia: a case control study. Ophthalmology. 2025;132:164–9. 39111668 10.1016/j.ophtha.2024.07.033

[10] Kim YG Oh JW Lee KC Yoon SH. Clinical association between serum cholesterol level and the size of xanthelasma palpebrarum. Arch Craniofac Surg. 2022;23:71–6. 35526842 10.7181/acfs.2022.00185 PMC9081426

[11] Gondane S Meherda A Kothiwala R. To study the prevalence of metabolic syndrome and dyslipidemia in patients of xanthelasma palpebrarum at a tertiary care hospital. Asian J Diabetol. 2020;21:10–4.

[12] Rai A Karki S Sah SP Kamat LN Pradhan M. Dyslipidemia in patients with xanthelasma palpebrarum visiting the Department of Dermatology of a tertiary care centre: a descriptive cross-sectional study. J Nepal Med Assoc. 2022;60:529–32. 10.31729/jnma.7485 PMC9275457 35690977

[13] Ma T Zhang J Li X. Investigation on 7 items of blood lipid in 1104 patients with eyelid xanthoma. South China J Cardiovasc Dis. 2022;28:256–9.

[14] Ma T Liu JY Li X. Attribution analysis of the correlation between xanthelasma palpebrarum and 7 serum lipid parameters among staff in Xingtai City. Knowl Cardiovasc Dis Prev Treat. 2022;12:28–30.

[15] Ozdöl S Sahin S Tokgözoğlu L. Xanthelasma palpebrarum and its relation to atherosclerotic risk factors and lipoprotein (a). Int J Dermatol. 2008;47:785–9. 18717856 10.1111/j.1365-4632.2008.03690.x

[16] Zhimin W Hui W Fengtao J Wenjuan S Yongrong L. Clinical and serum lipid profiles and LDLR genetic analysis of xanthelasma palpebrarum with nonfamilial hypercholesterolemia. J Cosmet Dermatol. 2020;19:3096–9. 32176424 10.1111/jocd.13366

[17] Li YR Wang ZM Wang H Yang YQ Ji FT. Study on single nucleotide polymorphisms of macrophage scavenger receptor 1 gene in xanthelasma palpebrarum patients. Int Eye Sci. 2019;19:1986–8.

[18] Hu W Liu Y Lian C Lu H. Genetic insight into ptative causes of xanthelasma palpebrarum: a Mendelian randomization study. Front Immunol. 2024;15:1347112. 38601164 10.3389/fimmu.2024.1347112 PMC11004296

[19] Wang J Huang CM Tang HY Dai DX Liu YC Chen YM. Study on the correlation between xanthelasma palpebrarum and the genetic factor of hypercholesterolemia. Int Eye Sci. 2023;23:689–93.

[20] Zhang MM Wang YQ. Etiology, characteristics and treatment progress of xanthoma palpebrarum. Chin J Aesthetic Med. 2023;32:194–8.

[21] Platsidaki E Kouris A Agiasofitou E Antoniou C Kontochristopoulos G. Periorbital hyperpigmentation in patients with xanthelasma palpebrarum: an interesting observation. J Clin Aesthet Dermatol. 2016;9:52– 4. 27721911 PMC4898585

[22] Nair PA Singhal R. Xanthelasma palpebrarum – a brief review. Clin Cosmet Investig Dermatol. 2018;11:1–5. 10.2147/CCID.S130116 PMC5739544 29296091

[23] Khode S Tan SHT Tan EA Uppal S. Xanthelasma palpebrarum: more than meets the eye. Indian J Otolaryngol Head Neck Surg. 2019;71(Suppl 1):S439–46. 10.1007/s12070-018-1345-0 PMC6848656 31742000

[24] Goyal P Gupta V Aithal S Meena D Dhillon KS. Assessment and predictors of metabolic syndrome in patients of xanthelasma palpebrarum: an observational study. Dermatol Pract Concept. 2024;14:e2024218. 39652943 10.5826/dpc.1404a218 PMC11619980

[25] Agarwal K Saikia P Podder I. Metabolic syndrome and dyslipidemia in xanthelasma palpebrarum and associated risk – 2 factors – a case-control study. J Cosmet Dermatol. 2022;21:7018–24. 36057448 10.1111/jocd.15353

